# The Human Minor Histocompatibility Antigen1 Is a RhoGAP

**DOI:** 10.1371/journal.pone.0073962

**Published:** 2013-09-23

**Authors:** Bart-Jan de Kreuk, Antje Schaefer, Eloise C. Anthony, Simon Tol, Mar Fernandez-Borja, Dirk Geerts, Jos Pool, Lothar Hambach, Els Goulmy, Peter L. Hordijk

**Affiliations:** 1 Department of Molecular Cell Biology, Sanquin Research and Landsteiner Laboratory, Academic Medical Center, Swammerdam Institute for Life Sciences, University of Amsterdam, Amsterdam, The Netherlands; 2 Department of Pediatric Oncology/Hematology, Erasmus University Medical Center, Rotterdam, The Netherlands; 3 Department of Immunohematology and Blood Transfusion, Leiden University Medical Center, Leiden, The Netherlands; 4 Department of Hematology, Hemostaseology, Oncology and Stem Cell Transplantation, Hannover Medical School, Hannover, Germany; Children's Hospital Boston, United States of America

## Abstract

The human minor Histocompatibility Antigen HMHA-1 is a major target of immune responses after allogeneic stem cell transplantation applied for the treatment of leukemia and solid tumors. The restriction of its expression to hematopoietic cells and many solid tumors raised questions regarding its cellular functions. Sequence analysis of the HMHA-1 encoding HMHA1 protein revealed the presence of a possible C-terminal RhoGTPase Activating Protein (GAP) domain and an N-terminal BAR domain. Rho-family GTPases, including Rac1, Cdc42, and RhoA are key regulators of the actin cytoskeleton and control cell spreading and migration. RhoGTPase activity is under tight control as aberrant signaling can lead to pathology, including inflammation and cancer. Whereas Guanine nucleotide Exchange Factors (GEFs) mediate the exchange of GDP for GTP resulting in RhoGTPase activation, GAPs catalyze the low intrinsic GTPase activity of active RhoGTPases, resulting in inactivation. Here we identify the HMHA1 protein as a novel RhoGAP. We show that HMHA1 constructs, lacking the N-terminal region, negatively regulate the actin cytoskeleton as well as cell spreading. Furthermore, we show that HMHA1 regulates RhoGTPase activity *in vitro* and *in vivo*. Finally, we demonstrate that the HMHA1 N-terminal BAR domain is auto-inhibitory as HMHA1 mutants lacking this region, but not full-length HMHA1, showed GAP activity towards RhoGTPases. In conclusion, this study shows that HMHA1 acts as a RhoGAP to regulate GTPase activity, cytoskeletal remodeling and cell spreading, which are crucial functions in normal hematopoietic and cancer cells.

## Introduction

Human minor Histocompatibility (H) antigens are important immunological barriers after allogeneic stem cell transplantation (SCT) applied for the treatment of leukemia and solid tumors. Minor H antigens are HLA-restricted peptides generated from specific intracellular polymorphic proteins. Upon presentation of these peptides on the cell surface, minor H antigens can stimulate cytotoxic T cells (CTLs) targeting these epitopes [1]. HMHA-1 was the first autosomal minor H antigen identified and is the most studied minor H antigen to date [2]. HMHA-1 CTLs can be detected frequently after allogeneic SCT coinciding with the Graft versus Leukemia effect [3]. HMHA-1 gene expression is restricted to the hematopoietic system, comprising normal and leukemic cells, including progenitor cells [4]. Although absent in normal epithelial cells, HMHA-1 gene expression is also observed in epithelial tumors of many different entities [5], suggesting a role of HMHA-1 in carcinogenesis [6]. Indeed, HMHA-1 CTLs eradicate human leukemia and solid tumors in immunosuppressed mice [7], [8]. The apparent relevance of HMHA-1 as antigen in the context of allogeneic SCT, the restriction of its expression to hematopoietic cells and its aberrant expression in solid tumors raised questions regarding its cellular functions.

Sequence analysis of the HMHA-1 encoding HMHA1 protein revealed the presence of a possible C-terminal RhoGTPase Activating Protein (GAP) domain and an N-terminal Bin/Amphiphysin/Rvs (BAR) domain [6]. Rho-family GTPases and in particular Rac1, Cdc42, and RhoA are key regulators of the actin cytoskeleton, which is dynamically remodeled during cell adhesion, spreading and migration [9]. These GTPases control cell morphology, polarity, cell adhesion and directional motility by regulating the formation of filopodia, lamellipodia, stress fibers, and focal adhesions in a tightly controlled fashion [10], [11]. When RhoGTPases are in their active, GTP-bound state, downstream effectors, such as the p21-activated kinase (PAK) serine/threonine kinase for Rac1, or Rho-associated coiled-coil-containing protein kinase (ROCK) [12] for RhoA, are activated to regulate downstream signaling.

Both activation and inactivation of RhoGTPases is under tight control. In response to extracellular stimuli, GEFs control the exchange of GDP for GTP, activating the GTPases [13]. Conversely, GAPs regulate the hydrolysis of GTP to GDP by catalyzing the low intrinsic GTPase activity, thereby inactivating the GTPase [14]. Finally, whereas most active RhoGTPases are associated to the inner leaflet of the plasma membrane, inactive RhoGTPases reside in the cytoplasm bound to the Rho guanine nucleotide dissociation inhibitor (RhoGDI) [15].

BAR domains are modules involved in membrane dynamics including endocytosis and vesicle transport [16], [17]. Many BAR domain-containing proteins have been shown to regulate RhoGTPase activity and function [18]. One subclass of these, including SH3BP1, Oligophrenin-1 (OPHN1), and GRAF1, [19]–[21] bear structural similarity to HMHA1 in that they encode both a BAR- as well as a GAP-domain. As the cellular role of HMHA-1 encoding HMHA1 protein is unknown we decided to investigate the biological function of HMHA1.

We show that ectopic expression of HMHA1 mutants lacking the N-terminal BAR domain but encoding the GAP domain dramatically alters the organization of the F-actin cytoskeleton. This is apparent from an overall loss of F-Actin as well as of focal adhesions which is accompanied by strongly impaired cell adhesion and spreading. We also show that HMHA1 interacts and colocalizes with different RhoGTPases. Both *in vitro* and *in vivo* studies showed that HMHA1 regulates RhoGTPase activity. Finally, we demonstrate that the HMHA1 BAR domain auto-inhibits its GAP function. In summary, our data identify HMHA1 as a novel RhoGAP which regulates actin dynamics and cell spreading.

## Materials and Methods

### Antibodies, Reagents, and Expression constructs

#### Antibodies

Anti-Actin (A3853), anti-α-Tubulin (T6199), anti-HA (H3663), and anti-HMHA1 (HPA019816) were from Sigma. Anti-c-myc (13–2500) was from Invitrogen. Anti-Paxillin (610620) was from Transduction Laboratories. For immunofluorescence, anti-Rac1 (05–389) was from Millipore, and for Western blot anti-Rac1 (610651) was from Transduction Laboratories. Secondary HRP-labelled antibodies for Western blot were from Pierce. Secondary Alexa-labelled antibodies for immunofluorescence were from Invitrogen. F-Actin was detected using Bodipy 650/665- Texas-Red- or Alexa-633-labelled Phalloidin (Invitrogen).

#### Expression constructs

To generate myc-tagged HMHA1 deletion constructs, pcDNA-2x-myc-HMHA1 was used as a template for PCR. The following primers were used: For myc-HMHA1 N-term, forward primer 5′-GAGATCGATATCAAGCTTTTCTCCAGGAAGAAACGAG-3′ and reverse primer 5′-GAGATCTCTAGAGGATCCTCAGGCCGCCTTGGACAGC-3′. For myc-HMHA1 C1-GAPtail, forward primer, 5′-GAGATCGATATCAAGCTTTTCCGCCACGAGGGGC-3′ and reverse primer 5′-GAGATCTCTAGAGGATCCTCACACGAATTCCGGCTGCC-3′. For myc-HMHA1 C1-GAP, forward primer 5′-GAGATCGATATCAAGCTTTTCCGCCACGAGGGGC-3′ and reverse primer 5′-GAGATCTCTAGAGGATCCTCAGCCGTAGTGGACGATG-3′. For myc-HMHA1 GAPtail, forward primer 5′-GAGATCGATATCAAGCTTCAGCTGTTCGGCCAGG-3′ and reverse primer 5′-GAGATCTCTAGAGGATCCTCACACGAATTCCGGCTGCC-3′. For myc-HMHA1 GAP, forward primer 5′-GAGATCGATATCAAGCTTCAGCTGTTCGGCCAGG-3′, and reverse primer 5′-GAGATCTCTAGAGGATCCTCAGCCGTAGTGGACGATG-3′. The products were cloned into a pcDNA-2x-myc vector. To generate GST-HMHA1 constructs, pcDNA-2x-myc-HMHA1 was used as a template for PCR. The following primers were used: For GST-HMHA1 FL, forward primer 5′-GAGATCGGATCCTTCTCCAGGAAGAAACGAG-3′ and reverse primer 5′-GAGATCTCTAGAGGATCCTCACACGAATTCCGGCTGCC-3′. For GST-HMHA1 N-term, forward primer 5′-GAGATCGGATCCTTCTCCAGGAAGAAACGAG-3′ and reverse primer 5′-GAGATCGCGGCCGCTCAGGCCGCCTTGGACAGC-3′. For GST-HMHA1 C1-GAPtail, forward primer 5′-GAGATCGGATCCTTCCGCCACGAGGGGC-3′ and reverse primer 5′-GAGATCTCTAGAGGATCCTCACACGAATTCCGGCTGCC-3′. The product was cloned into pGex-6p-1. All fusion constructs were confirmed by sequencing. pmCherry(C1) was from Clontech Laboratories. mCherry-Rac1 Q61L and G12V were described previously (De Kreuk et al., 2011). HA-tagged RhoA V14 and Cdc42 G12V were purchased from Missouri S&T cDNA Rescource Center. GST-Rac1 WT was described previously [22]. GST-Rac1ΔC and GST-RhoAΔC were a kind gift from A. Wittinghofer (Max-Planck Institute for Molecular Physiology, Dortmund, Germany).

### Lentiviral shRNAi and siRNA silencing

Lentiviral shRNA constructs for HMHA1 from the TRC/Sigma Mission library were obtained from Sigma-Aldrich (St. Louis, MI, USA). Scrambled shRNA (SHC002; Sigma-Aldrich) was used as a negative control. Lentiviral particles expressing shRNA constructs were prepared using HEK293T cells and virus was transduced as described previously [23].

### SDS-PAGE and Western blot analysis

Proteins were separated on SDS-PAGE gels followed by transfer onto nitrocellulose transfer membrane using the iBlot Dry Blotting System (Invitrogen) according to the manufacturers' recommendations. After blotting, membranes were blocked in 5% low fat milk in TBST (Tris-Buffered Saline Tween-20) for 30 minutes and subsequently the blots were incubated with the primary antibody overnight at 4°C. Next, the blots were washed 3 times for 30 minutes in TBST and subsequently incubated with HRP-conjugated secondary antibodies in TBST for 1 hour at RT. Finally, blots were washed 3 times with TBST for 30 minutes each. Blots were developed by ECL (GE Healthcare, Hoevelaken, The Netherlands).

### Confocal Laser Scanning Microscopy and FACS analysis

Twenty-four hours after cells were seeded on fibronectin-coated glass coverslips, the indicated plasmids were transfected. After 24 hours, cells were fixed by 3.7% formaldehyde (Merck) in PBS (10 minutes; RT) followed by permeabilization with 0.5% Triton X-100 in PBS (5 minutes; RT). Coverslips were then incubated for 15 minutes with 2% BSA in PBS at 37°C to prevent aspecific binding. Immunostainings were performed with the indicated antibodies (60 minutes; RT). Fluorescent imaging was performed with a confocal laser scanning microscope (LSM510/Meta; Carl Zeiss MicroImaging, Inc.) using a 63X/NA 1.40 (Carl Zeiss MicroImaging, Inc.). Image acquisition was performed with Zen 2009 software (Carl Zeiss MicroImaging, Inc.). Image analysis for quantification of paxillin staining was further performed using Image J. Here, the average pixel intensity of paxillin staining, after background subtraction and correction for surface area, was calculated for 10–20 cells per condition. The mean +/− SD was calculated and statistical differences were determined using a students' t-test.

For FACS and image analysis using the Image Stream technology (Amnis), Jurkat T cells were treated or not with 100 ng/ml CXCL12 (300-28A, Peprotech) for the indicated time-points, fixed and permeabilized using Intraprep fix and perm (IM2388, Beckman Coulter). Cells were subsequently stained for HMHA1 and Rac1 and for F-actin using Bodipy-labelled phalloidin. Image Stream analysis software was used for processing of the data.

### Cell culture and transfections

Jurkat and HeLa cells were maintained at 37°C and 5% CO_2_ in Iscove's Modified Dulbecco's Medium (IMDM; Biowhittaker) suplemented with 10% heat-inactivated Fetal Calf Serum (Life Technologies, Breda, The Netherlands), 300 µg/ml glutamine, 100 units/ml penicillin and streptomycin. HeLa cells were transiently transfected with T*rans*IT (Mirus) according to the manufacturers' recommendations.

### GST Pull-Down Assays

For studying the direct interaction of HMHA1 with Rac1 or RhoA, GST-fusion proteins were purified from BL21 bacteria as described previously [22]. GST-HMHA1 was cut with precision protease (GE Healthcare) overnight at 4°C while rotating. Next, supernatant, containing purified HMHA1 without GST-tag and beads, was harvested and used for the interaction studies or *in vitro* GAP assay. GST-Rac1 and RhoA were allowed to bind GDP or GppNHP overnight at 4°C while rotating. Binding of HMHA1 to the RhoGTPases was assayed by Western blot analysis using the anti-HMHA1 antibody.

### RhoGTPase activity assays

Rac1 activation in HeLa or Jurkat cells, transfected/transduced as indicated, was analyzed by a CRIB-peptide pull-down approach as described previously [22]. Cells were lysed in NP-40 lysis buffer (50 mM TRIS/HCl pH 7.5, 100 mM NaCl, 10 mM MgCl_2_, 10% glycerol and 1% NP40) supplemented with protease inhibitors (Complete mini EDTA, Roche). Subsequently, lysates were centrifuged at 20.000 xg for 10 minutes at 4°C. The supernatant was then incubated with 30 µg of Pak1-CRIB peptide and incubated at 4°C for 1 hour while rotating. Bound Rac1GTP levels were detected by Western blot analysis.

Levels of RhoAGTP were measured using a RhoA G-Lisa kit (BK124; Cytoskeleton) according to the manufacturers' recommendations.

*In vitro* GAP activity of HMHA1 was measured using a RhoGAP Assay (BK105; Cytoskeleton) according to the manufacturers' recommendations. In short, purified HMHA1 protein (see above) was incubated together with the small GTPases, Rac1, Cdc42, RhoA, and Ras in the presence of GTP (20 minutes; 37°C). Free inorganic phosphate (generated by the hydrolysis of GTP to GDP) was detected by CytoPhos and subsequently absorbance (650 nm) was measured. We used GTPase or GAP protein only as a negative control and as a measure for the intrinsic hydrolysis rate. p50RhoGAP was used as a positive control for the assay.

### Electric resistance measurements

For ECIS-based cell spreading experiments, golden ECIS electrodes (8W10E; Applied Biophysics) were treated with 10 µM L-cysteine for 15 minutes. Subsequently electrodes were coated with 10 µg/ml fibronectin in 0.9% NaCl for 1 hour at 37°C. Next, HeLa cells, transfected as indicated, were seeded at a concentration of 100.000 cells per well in 400 µl IMDM with 10% FCS. Impedance was measured continuously at 45 kHz using ECIS model 9600. The increase in impedance, as a measure of cell spreading [24], was recorded for one hour.

### Homology Modeling

The homology model of the HMHA1 RhoGAP domain was calculated by submitting the sequence of the human HMHA1 RhoGAP domain (residues 753–973) to the Phyre protein structure prediction server which includes sequence alignments with several RhoGAPs [25]. Superimpositions and figures were prepared with PyMOL (PyMOL Molecular Graphics System, Schroedinger, LLC).

## Results

### HMHA1 regulates the actin cytoskeleton and cell spreading

**Figure 1:**
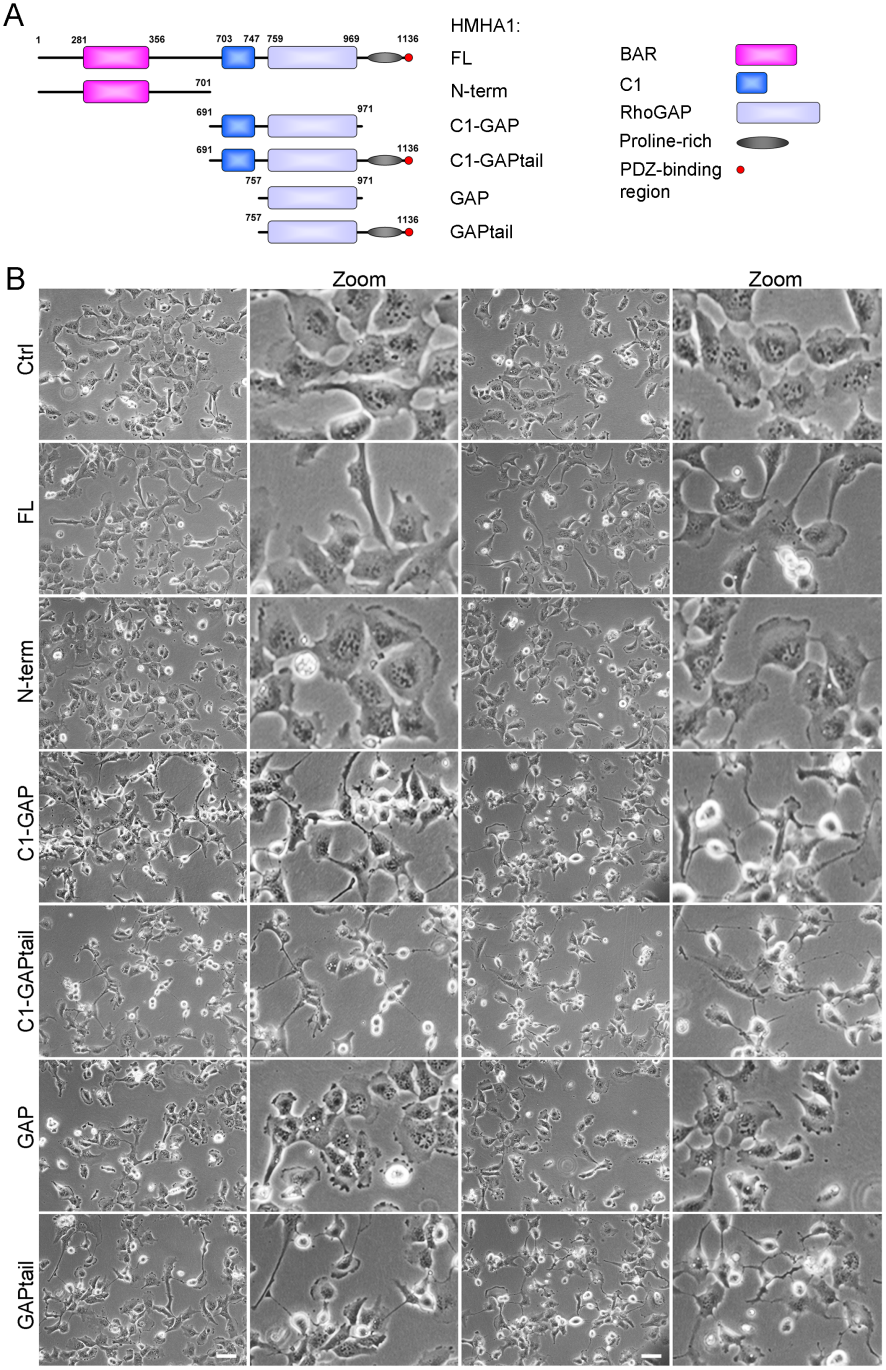
HMHA1 mutants and morphological effects. (A) Schematic overview of the organization of HMHA1 and of the different constructs used in this study. (B) Morphology of HeLa cells, transfected as indicated, was analyzed by phase contrast microscopy. Cells expressing HMHA1 full-length (FL), GAP, or N-term did not show any changes in morphology compared to control cells. HMHA1 C1-GAP, C1-GAPtail, and GAP-tail induce dramatic changes in cell morphology. In addition, these cells are less adhesive than control cells. Scale bars, 50 µm.

Analysis of the HMHA1 protein sequence shows that it encodes an N-terminal BAR domain followed by a C1 domain and a RhoGAP domain. The C-terminal portion of the protein consists of a proline-rich region as well as a PDZ-binding domain (Figure 1A). Interestingly, similar to HMHA1, other BAR domain proteins such as OPHN1 and GRAF1, also encode a RhoGAP domain [18] and are involved in regulating the actin cytoskeleton. We therefore investigated the role of HMHA1 in RhoGTPase signaling and actin remodeling.

We generated immunotagged full-length and deletion constructs of HMHA1 (Figure 1A). Because in several BAR-GAP proteins the BAR domain autoinhibits GAP function [26], both an N-terminal BAR-domain-containing construct and C-terminal constructs including the RhoGAP domain were generated (Figure 1A).

In addition to its hematopoietic specific expression (10), the HMHA1 gene is also expressed in epithelial tumor cells [5]. This interesting dual expression challenged us to visualize HMHA1 cellular protein distribution and dynamics. Hereto, different HMHA1 constructs were expressed in HeLa cells that do not express HMHA1 endogenously. We found no effects of full-length HMHA1 (FL) or the N-terminal construct (N-term) on overall morphology of HeLa cells (Figure 1B, upper three rows). Interestingly, cells expressing the C1-GAP, the C1-GAPtail and the GAPtail proteins show reduced membrane ruffling and extensive formation of cellular spines. Additionally, these cells show reduced cell spreading (Figure 1B). Our results also show that the GAP domain of HMHA1 induces a significant change in cell shape. The finding that FL HMHA1 does not induce this phenotype, is suggestive for a negative regulatory role of the BAR domain.

**Figure 2:**
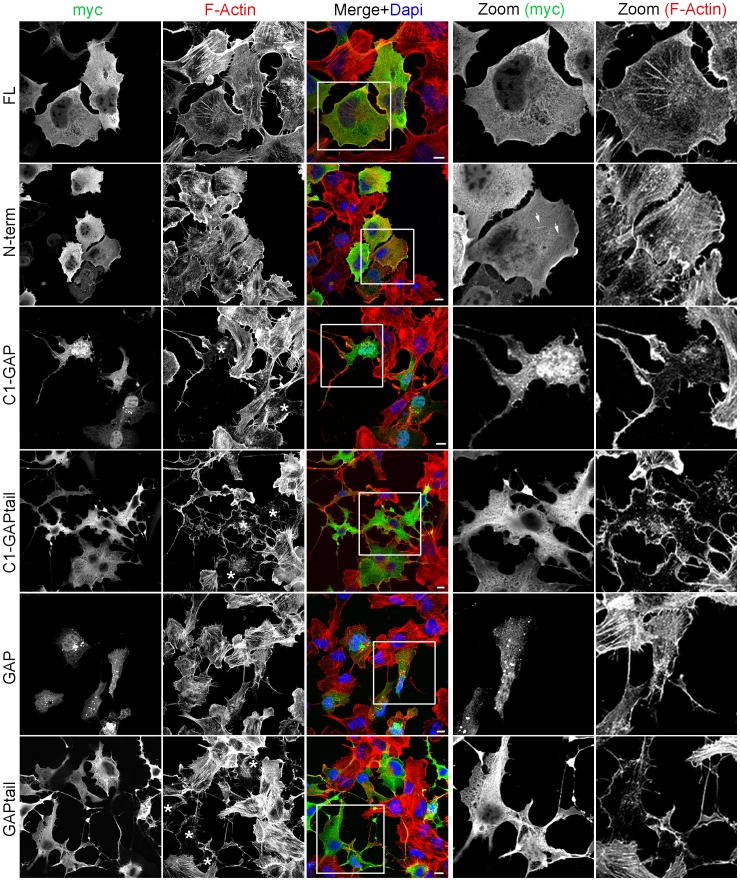
Localization and effects on F-actin of HMHA1 and selected mutants. Intracellular localization of myc-tagged HMHA1 (and mutant constructs) was studied by Confocal Laser Scanning Microscopy following expression in HeLa cells. Myc-tagged HMHA1 was detected by immunostaining for the myc epitope in combination with detection of F-Actin with phalloidin. Full-length HMHA1 (FL) as well as HMHA1 N-term are partially localized at membrane ruffles as well as in the cytoplasm. For HMHA1 N-term localization at vesiculo-tubular structures is occasionally observed (arrows). Cells expressing FL or the N-term constructs are morphologically similar to control cells and no effects are seen on F-Actin (upper two rows). HMHA1 constructs lacking the C-terminal tail (GAP and C1-GAP) are partly mislocalized into protein aggregates. In cells expressing HMHA1 C1-GAP, C1-GAPtail, and GAPtail (marked with asteriks), F-Actin distribution is altered and cell morphology is dramatically changed. Higher magnification images of the boxed area are included. Scale bars, 10 µm.

Full-length (FL) HMHA1 localized to the cytoplasm as well as to membrane ruffles (Figure 2). A similar distribution was found for the N-terminal (N-term) construct, encoding the BAR domain. Interestingly, a fraction of HMHA1-N-term localizes to tubular structures (Figure 2) known to be formed by the membrane-deforming activity of BAR domains. The HMHA1 C1-GAPtail or GAPtail proteins show primarily a diffuse cytoplasmic distribution with a small fraction detectable on the plasma membrane (Figure 2). Interestingly, expression of HMHA1 C1-GAP and GAP results in formation of protein aggregates suggesting that the C-terminal tail-region is (partially) involved in proper localization of the HMHA1 protein (Figure 2).

Because the actin cytoskeleton is under tight control of RhoGTPases, we examined F-actin organization in cells expressing the different HMHA1 mutants. Neither the HMHA1 (FL) nor the N-terminal construct affect F-Actin distribution (Figure 2; upper two rows). However, constructs lacking the N-terminal BAR domain (C1-GAPtail, C1-GAP, GAP, GAPtail) induce a dramatic loss of F-actin (Figure 2; bottom 4 rows). In contrast to what we observed for F-actin, the microtubule network remains unaffected in cells expressing the different N-terminal deletion constructs (Figure S1).

**Figure 3:**
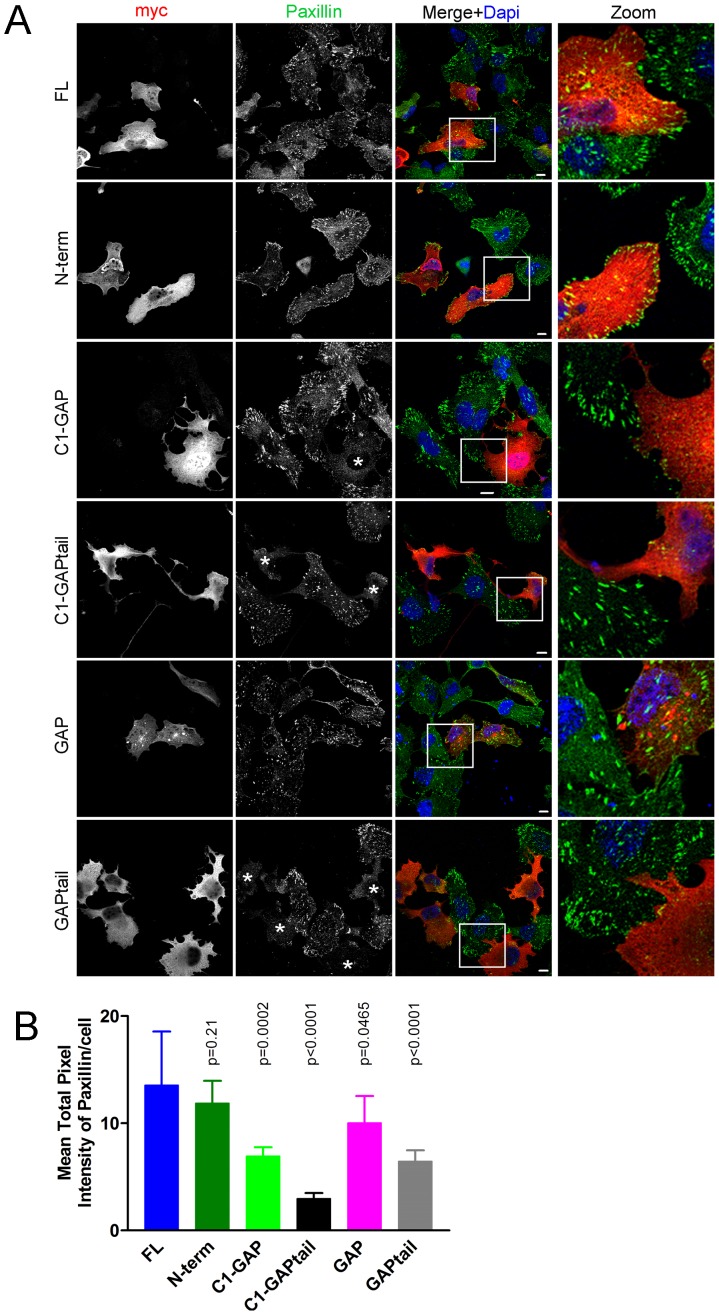
The HMHA1 GAP domain induces loss of focal adhesions. The effects of myc-tagged HMHA1 (and deletion constructs) on focal adhesion distribution was studied by Confocal Laser Scanning Microscopy following expression in HeLa cells. Similar to the effects on F-Actin distribution, cells expressing full-length HMHA1 (FL), N-term, or GAP (first, second and fifth rows) constructs show normal focal adhesion distribution as detected using Paxillin immunostainings. Expression of HMHA1 C1-GAP, C1-GAPtail, or GAPtail (marked with asteriks) induces loss of focal adhesions. In the merged images, HMHA1 constructs appear in red, paxillin in green and nuclei in blue. Higher magnification images of the boxed area are included. Scale bars, 10 µm. (B) Mean +/− SD of the average-per-cell paxillin staining intensity (10–20 cells per condition), quantified following background subtraction, is indicated. Statistical differences compared to the Full-Length control are indicated by the respective *p*-values.

We analyzed possible effects on integrin-mediated adhesion by recording focal adhesion distribution. Focal adhesions were visualized by immunostaining for paxillin [27]. Whereas HMHA1 FL and N-term proteins did not affect the distribution of focal adhesions, the C1-GAPtail, C1-GAP and GAPtail proteins induced a marked loss of focal adhesions (Figure 3A). Paxillin-positive structures were detected, albeit limited in number and very small, mainly at the periphery of the cells, suggestive for a defect in focal adhesion maturation. We therefore quantified focal adhesion density based on paxillin staining and using image analysis software (Figure 3B). These experiments indicate an important role for HMHA1 in regulation of the actin cytoskeleton, and in focal adhesion formation and distribution.

**Figure 4:**
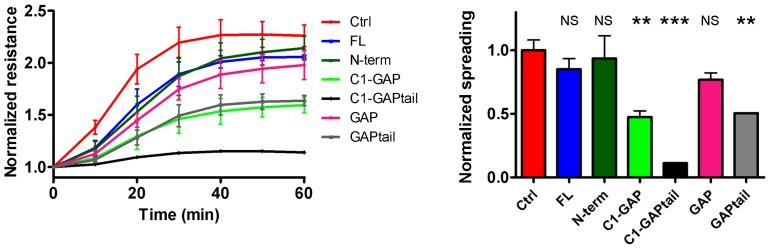
The HMHA1 GAP domain negatively affects cell spreading. Cell spreading was measured by Electrical Cell-substrate Impedance Sensing (ECIS) following seeding of 100.000 cells on fibronectin-coated electrodes. Left panel: A significant decrease in electrical resistance, as a measure of cell spreading, was observed in HeLa cells expressing HMHA1 C1-GAPtail (black), C1-GAP (light green), and GAPtail (grey) compared to control cells (red). Ectopic expression of HMHA1 full-length (blue), N-term (dark green), and GAP (magenta) did not affect cell spreading. Right panel: Relative cell spreading at 60 minutes post-seeding. Data are mean values of three independent experiments. Error bars indicate SEM. ns, not significant, ** p<0.01, *** p<0.001.

To confirm that cells expressing HMHA1 mutants lacking the N-terminal region are less adhesive, we analyzed cell spreading by ECIS (Electrical Cell-substrate Impedance Sensing) (Figure 4). In these assays, cells are seeded on golden electrodes and the increase in impedance, a measure for cell spreading, is recorded in real-time [24]. HMHA1 FL (blue curve) or N-term (dark green curve) did not significantly reduce cell spreading. However, cells expressing HMHA1 C1-GAPtail (black), C1-GAP (light green), and GAPtail (grey), showed a significant decrease in cell spreading. HMHA1 GAP (magenta) slightly reduced cell spreading, but this effect did not reach statistical significance (Figure 4).

These data suggest an important auto-inhibitory role for the HMHA1 BAR domain comparable to what was shown for other BAR-GAP proteins such as OPHN1 and GRAF1 [26], [28]. The HMHA1 GAP protein does not affect cell morphology or F-actin distribution to the extent we observed for the HMHA1 GAPtail or C1-GAP proteins, suggesting that either the C1 domain or the C-terminus, which encodes a proline-rich region and a PDZ-binding motif, is required for proper functioning of the HMHA1 RhoGAP domain.

### HMHA1 interacts and colocalizes with Rho-family GTPases

**Figure 5:**
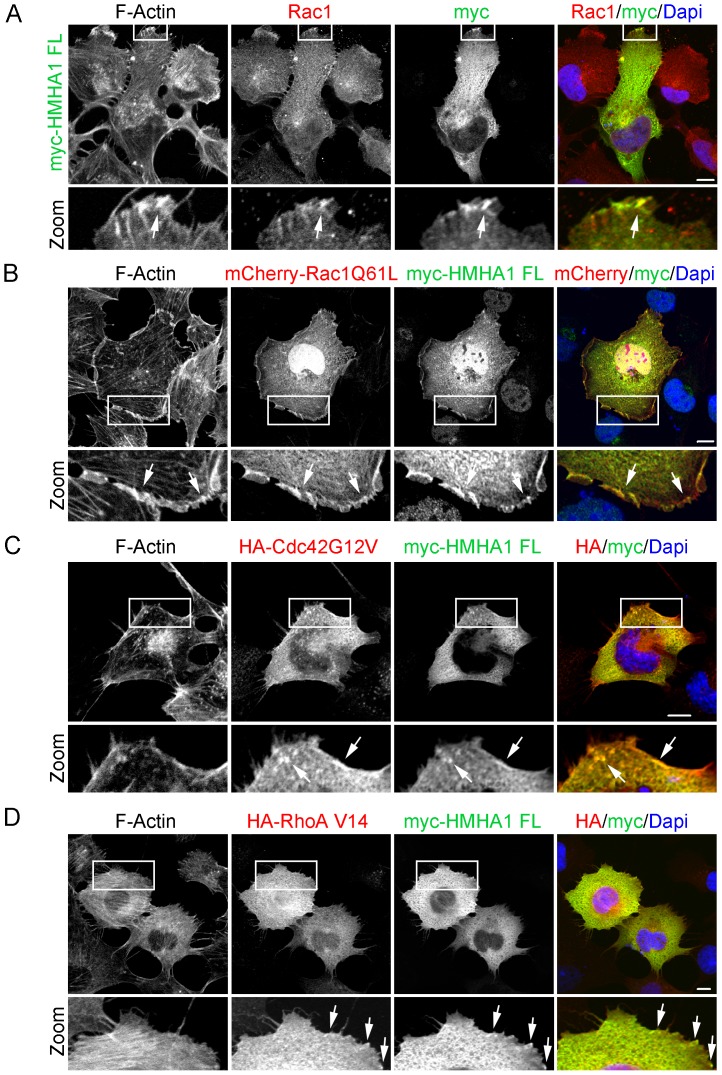
HMHA1 colocalizes with RhoGTPases. (A-D) Colocalization of myc-tagged HMHA1 with endogenous Rac1 (A), Rac1 Q61L (B), Cdc42 G12V (C) and RhoA V14 (D) was studied by Confocal Laser Scanning Microscopy. Myc-tagged HMHA1 and HMHA-tagged Cdc42 and RhoA were detected by immunostaining in combination with detection of F-Actin. HMHA1 colocalized with endogenous Rac1 (A) and Rac1 Q61L (B) in membrane ruffles (arrows). A partial colocalization of HMHA1 with Cdc42 G12V (C) and RhoA V14 (D) was observed (arrows) although less clear than for Rac1. Higher magnification images of the boxed areas are included. Scale bars, 10 µm.

Assuming that HMHA1 is putative RhoGAP, it is important to realize that RhoGTPases, in particular Rac1, Cdc42, and RhoA, are key regulators of actin reorganization. This knowledge led us to analyze whether HMHA1 co-localizes with these RhoGTPases. Interestingly, we found that HMHA1 co-localizes with Rac1 at sites with high actin dynamics such as peripheral membrane ruffles (Figure 5A). As GAP proteins are known to preferably bind to active RhoGTPases, full-length HMHA1 was cotransfected with mCherryRac1Q61L, a constitutively active Rac1 mutant. Similar to what we observed for endogenous Rac1, HMHA1 co-localizes with mCherryRac1Q61L in peripheral membrane ruffles (Figure 5B). In addition, we analyzed co-localization of full-length HMHA1 with the constitutively active mutants of Cdc42 (G12V; Figure 5C) or RhoA (V14; Figure 5D). We observed a limited co-localization of HMHA1 with Cdc42G12V and with RhoAV14 (Figure 5C, D; arrows).

**Figure 6:**
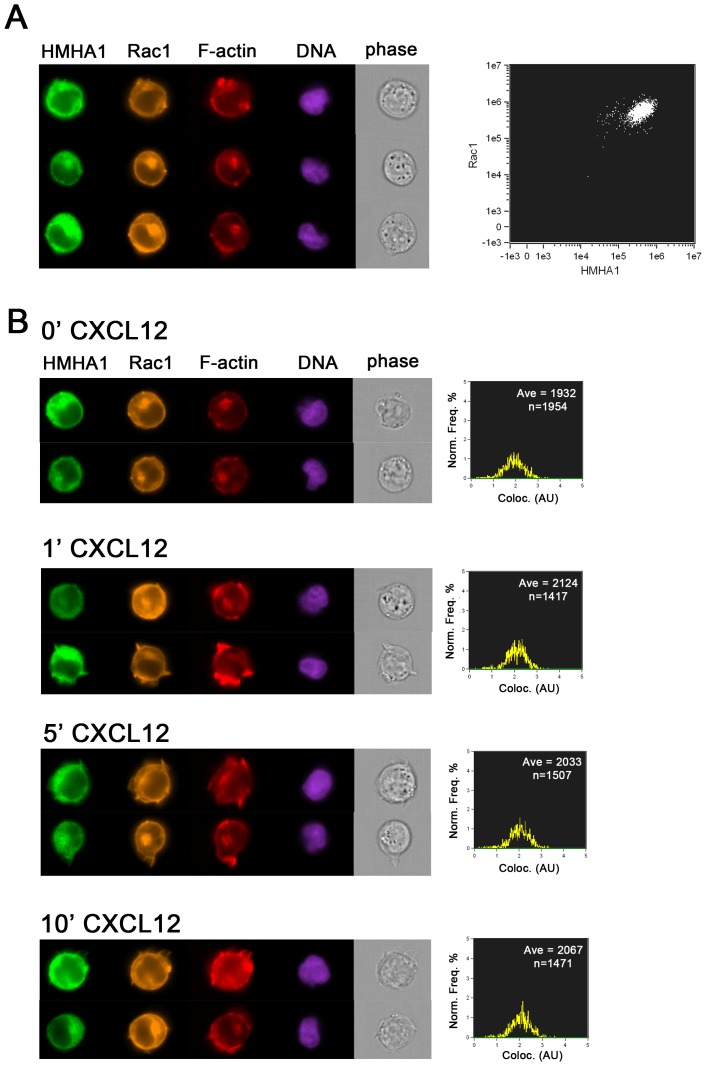
Visualization and flow cytometry analysis of endogenous HMHA1 using ImageStream. (A) Jurkat T-cells were fixed and immunostained for endogenous HMHA1 and Rac1 and stained for F-actin and DNA. Left panel shows three examples of the distribution of HMHA1, Rac1 and F-actin revealing colocalization of HMHA1 and Rac1 in F-actin rich areas. The nucleus (DNA) and cell morphology (phase image) are included to show the integrity of the cell. Right panel shows intensity distribution of Rac1 (Y-axis) and HMHA1 (X-axis) signals, underscoring the fact that most cells are double positive. (B) Jurkat cells were stimulated for the indicated time-points with 100 ng/ml CXCL12 and analyzed as in A. Two examples of each condition are shown in the left panels. Changes in F-actin distribution in response to CXCL12 can be observed, in particular after 1 and 5 minutes. Right panels show the extent of colocalization (AU, arbitrary units) quantified by the image stream software. Ave, average colocalization, n, number of cells.

To investigate this further for endogenous proteins, we immunostained Jurkat T–cells with antibodies to HMHA1 and Rac1 and analyzed the extent of colocalization by FACS analysis combined with microscopic imaging. This was done using the Image Stream technology that allows high-throughput, quantitative image analysis of a large number of cells. The data in Figure 6A show that endogenous HMHA1 and Rac1 show a high level of colocalization and that both proteins also localize to peripheral, F-actin-rich areas. The controls for the signal selectivity in the different channels are in Figure S2. To investigate if this localization is subject to regulation by extracellular stimuli, we treated cells for several time-points with the chemokine CXCL12. Subsequent FACS and image analysis (Figure 6B) confirmed induction of F-actin dynamics after brief stimulation with CXCL12 [29] and concomitant quantitative analysis of co-localization of HMHA1 and Rac1 showed that there is a transient, small increase in colocalization in response to CXCL12. However, this transient effect is limited, likely due to the already high level of colocalization of these two proteins. Both Rac1 and HMHA1 followed the CXCL12-induced actin dynamics and remained localized to peripheral F-actin rich areas.

We next performed pull-down experiments with GST-Rac1 (used as a model for RhoGTPases) and determined the direct interaction with bacterially purified HMHA1 C1-GAPtail. Our data show that HMHA1 C1-GAPtail interacts with Rac1 preferably when Rac1 is loaded with GppNHp, a non-hydrolysable analog of GTP (Figure S3A). Previously, we identified several proteins that regulate Rac1 activity, such as PACSIN2 and caveolin, that interact with the Rac1 C-terminal hypervariable domain [22], [30]. To assess whether purified HMHA1 requires the Rac1 C-terminal hypervariable domain for association, we performed pull-down experiments with GST-fusions of Rac1WT and Rac1ΔC, which lacks the hypervariable domain, loaded with GDP or GppNHp. In contrast to PACSIN2 and caveolin, HMHA1 C1-GAPtail binds to Rac1, independent of the C-terminal hypervariable domain (Figure S3B). To test the specificity of this interaction, we performed pull-down experiments with GST-Rac1ΔC and GST-RhoAΔC loaded with either GDP or GppNHp. We found that purified HMHA1 directly interacts with both Rac1 and RhoA. In line with the above findings, HMHA1 preferably interacts with Rac1 and RhoA when they are in the active, GppNHp-bound, conformation (Figure S3C).

In summary, HMHA1 colocalizes with RhoGTPases at sites of high membrane dynamics and interacts directly with Rac1 and RhoA.

### Active Rac1 but not Cdc42 rescues the phenotype induced by HMHA1 C1-GAPtail

**Figure 7:**
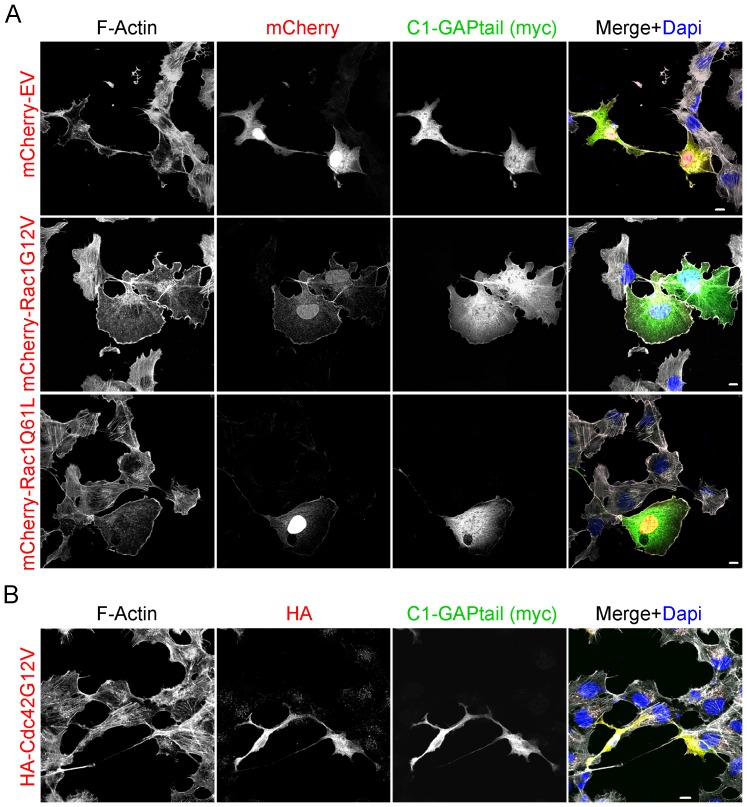
Constitutively active Rac1, but not Cdc42, rescues the altered cell morphology induced by HMHA1 C1-GAPtail. (A,B) Rescue experiments with constitutively active Rac1 Q61L and G12V (A) or Cdc42 G12V (B), co-expressed with the HMHA1 C1-GAPtail protein were performed in HeLa cells and analyzed by Confocal Laser Scanning Microscopy. Ectopically expressed proteins were visualized in combination with F-Actin. (A) Constitutively active Rac1 Q61L (middle panels) and G12V (bottom panels) were able to (partially) rescue the phenotype induced by C1-GAPtail. As a control, mCherry empty vector (EV; upper panels) was unable to rescue the phenotype. (B) Ectopic expression of constitutively active Cdc42 G12V did not rescue the phenotype induced by C1-GAPtail. Scale bars, 10 µm.

Next we investigated whether ectopic expression of constitutively active mutants of Rac1 (Rac1G12V and Q61L) or Cdc42 (G12V), that are unresponsive to GAP activity, could rescue the phenotype induced by HMHA1 C1-GAPtail. Interestingly, both mCherry-Rac1Q61L and G12V were able to bypass the effect induced by HMHA1 C1-GAPtail (Figure 7A) in that cells expressing both constitutively active Rac1 as well as HMHA1 C1-GAPtail show a more spread phenotype with less spine-like protrusions as compared to the controls (mCherry-EV) (Figure 7A). In contrast to active Rac1 mutants, Cdc42G12V did not rescue cell morphology and spreading induced by the C1-GAPtail protein (Figure 7B). We could not test whether constitutively active RhoA (V14) rescued cell morphology or -spreading since both active RhoA and HMHA1 C1-GAPtail induce contracted and loosely-adherent cells. Likely as a result of this phenotype, we could not detect any double-positive cells. Therefore, whether activation of RhoA could rescue the dramatic phenotype induced by C1-GAPtail remains unclear.

### HMHA1 is a RhoGAP *in vitro* and its GAP function is inhibited by the BAR domain

**Figure 8:**
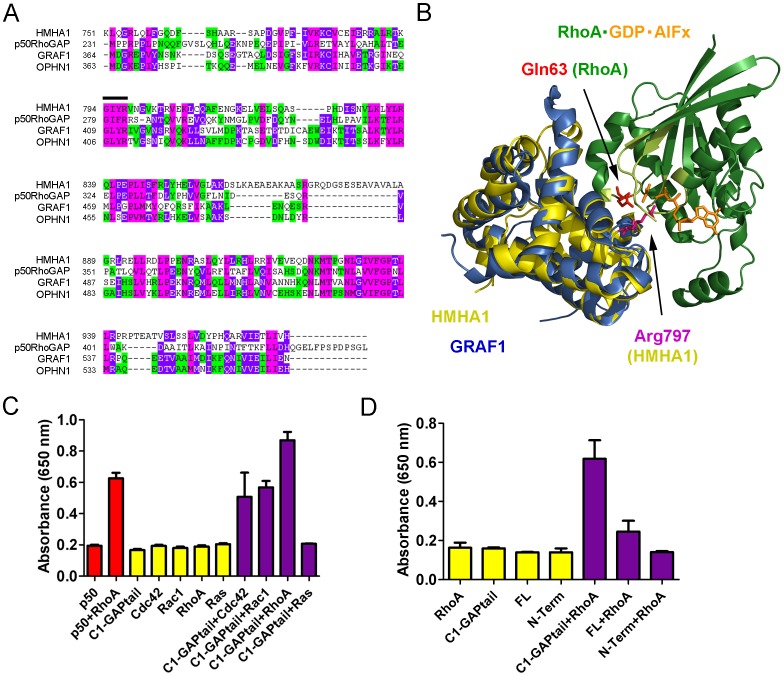
HMHA1 is a RhoGAP *in vitro*. (A) Sequence alignment of HMHA1 with the typical RhoGAP, p50RhoGAP, and the structurally-related BAR-GAPs, GRAF1 and OPHN1. Green indicates two matching amino acids. Pink indicates three matching amino acids. Purple indicates four matching amino acids. The arginine finger region is indicated with a black bar. (B) 3D model of the protein-protein complex between RhoA and the HMHA1 RhoGAP domain highlighting the catalytic residues (in sticks, colour coding as indicated; P-loop-Switch I-Switch II of RhoA in light green). The homology model for the GAP domain of human HMHA1 is based on the structure of the human p50RhoGAP domain (PDB ID: 1tx4), using Phyre. The position of the HMHA1 GAP domain in the complex with human RhoA (from RhoA⋅GDP⋅AlFx⋅p50RhoGAP; PDB ID: 1tx4) was obtained through its overlay on the p50RhoGAP domain. The RhoGAP domain of GRAF1 from *Gallus gallus* (PDB ID: 1f7c) was superimposed onto the model of the HMHA1 GAP domain. (C) HMHA1 C1-GAPtail has *in vitro* GAP activity towards Rac1, Cdc42, and RhoA but not towards Ras (purple bars). p50RhoGAP was used as a positive control (red bars). GTPases or HMHA1 only were used as a control and as a measure for intrinsic nucleotide hydrolysis (yellow bars). Data are mean values of two independent experiments. Error bars indicate SD. (D) HMHA1 GAP activity is inhibited by the N-terminal BAR domain as full-length HMHA1 has no GAP activity while C1-GAPtail, lacking the N-terminal region, shows GAP activity (purple bars). GTPases or HMHA1 only were used as a control and as a measure for intrinsic hydrolysis (yellow bars). Data are mean values of two independent experiments. Error bars indicate SD.

Next we analyzed the homology of the HMHA1 predicted RhoGAP domain in comparison to p50RhoGAP, a prototypical RhoGAP. Based on a similar BAR-GAP architecture we included GRAF1 and OPHN1 in the analysis. Sequence alignment clearly demonstrates high sequence homology of the HMHA1 RhoGAP domain with the different human RhoGAPs p50RhoGAP, GRAF1, and OPHN1, including a conserved Arg residue at position 797 (Figure 8A; black bar).

Next, we generated a homology model of the HMHA1 RhoGAP domain based on the structure of the human p50RhoGAP domain (Protein Data Bank (PDB) ID: 1tx4), the latter being the top-scoring model predicted by the Phyre protein structure prediction server [25]. Similar to other well-characterized RhoGAPs, including GRAF1 (in blue), the HMHA1 RhoGAP domain (in yellow) shows, with 9 α-helices, an exclusively helical structure (Figure 8B). A hallmark of RhoGAPs and other GAPs is the formation of a high-affinity complex with the cognate inactive GDP-bound GTPase, only in the presence of aluminium fluoride AlFx. This mimics the transition state of the GTP hydrolysis (Rho⋅GDP⋅Pi⋅RhoGAP) [31]. The position of the HMHA1 RhoGAP domain in complex with human RhoA bound to GDP⋅AlFx (from RhoA⋅GDP⋅AlFx⋅p50RhoGAP; PDB ID: 1tx4), was defined through its superposition onto the p50RhoGAP domain (Figure 8B). As described for other RhoGAPs, the HMHA1 GAP domain interacts mainly with the P-loop and the switch regions of RhoA (in light-green, Figure 8B). The invariant Arg797 of the HMHA1 RhoGAP domain which may represent the catalytic Arg residue (Arg finger) is orientated into the active site of RhoA, close to AlFx and the nucleotide phosphates (Figure 8B). The catalytic Arg residue neutralizes the developing charge during the GTP hydrolysis and thus stabilizes the transition state [31]–[33]. The highly conserved Gln residue in the switch II region of Rho GTPases (Gln63 in RhoA, Gln61 in Rac1/Cdc42) is required for an efficient GAP-catalyzed GTP hydrolysis as well, since it coordinates the attacking water for the GTP cleavage [31]–[33].

Our homology model indicates that HMHA1 contains the structural requirements to function as a GAP protein and to stimulate the GTP hydrolysis of Rho GTPases. To confirm this, we analyzed GAP activity of HMHA1 *in vitro* using purified proteins in a cell-free system. As a control, we measured GAP activity of p50RhoGAP towards RhoA. As expected, when combining p50RhoGAP with GTP-loaded RhoA, we observed an increase in the release of inorganic phosphate, generated upon GTP hydrolysis (Figure 8C; red bars). To analyze HMHA1 GAP activity we used the purified C1-GAPtail protein. These experiments showed that HMHA1 C1-GAPtail catalyzes GTP hydrolysis by Rac1, RhoA, and Cdc42 (Figure 8C; purple versus yellow bars). HMHA1 C1-GAPtail did not catalyze GTP hydrolysis by Ras (Figure 8C; right bar). These data indicate that HMHA1 acts as a RhoGAP *in vitro*.

Several BAR-GAP proteins, including OPHN1 and GRAF1, are autoinhibited by their BAR domain [26]. As HMHA1 is structurally similar to these proteins and because dramatic effects on actin dynamics and cell spreading are observed upon expression of HMHA1 constructs lacking the BAR domain, we tested whether the HMHA1 BAR domain auto-inhibits GAP function towards RhoGTPases. We performed *in vitro* GAP assays using purified RhoA and HMHA1 FL, as well as the N-terminal construct including the BAR domain (N-term), and HMHA1 C1-GAPtail. Similar to what has been shown for GRAF1 and OPHN1, full-length HMHA1 as well as HMHA1 N-term showed little or no GAP activity compared to HMHA1 C1-GAPtail which lacks the N-terminal BAR domain (Figure 8D) suggesting that HMHA1 is both structurally as well as functionally similar to GRAF1 and OPHN1. In summary, these experiments show that HMHA1 is a genuine RhoGAP and its GAP function is inhibited by the N-terminal BAR domain.

### HMHA1 regulates GTPase activity *in vivo*

**Figure 9:**
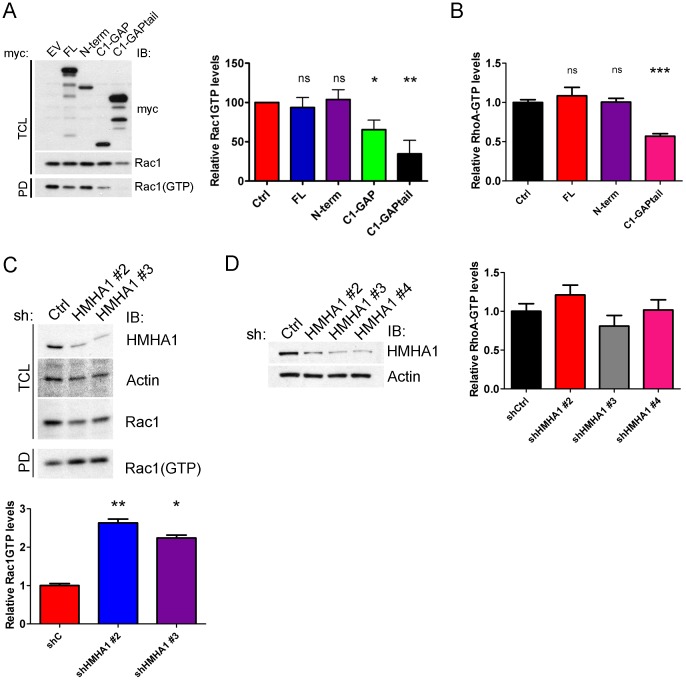
HMHA1 regulates RhoGTPase activity *in vivo*. (A) HeLa cells were transfected as indicated. After 24 hours, a CRIB pull-down assay was performed to measure levels of Rac1GTP. HMHA1 full-length (blue bar) and N-term (purple bar) do not significantly decrease Rac1GTP loading. The N-terminal BAR domain auto-inhibits GAP function as HMHA1 C1-GAP (green bar) and C1-GAPtail (black bar), which lack the N-terminal BAR domain, show a significant decrease in Rac1GTP loading compared to control cells (red bar). Data are mean values of five independent experiments. Error bars indicate SEM. ns, not significant. * p<0.05, ** p<0.01. (B) HeLa cells were transfected as indicated. After 24 hours, a RhoA G-LISA (Cytoskeleton) was used to measure levels of RhoA-GTP. HMHA1 full-length (red bar) and N-term (purple bar) did not significantly decrease RhoA-GTP loading. In contrast, HMHA1 C1-GAPtail (pink bar), which lacks the N-terminal BAR domain, showed a significant decrease in RhoA-GTP loading compared to control cells (red bar). Data are mean values of three independent experiments. Error bars indicate SEM. ns, not significant, * <0.05, *** p<0.001. (C) Jurkat cells were transduced with control (shC) or HMHA1 (shHMHA1 #2 or #3) short hairpin RNAs. After 72 hours, a CRIB pull-down assay was performed to measure levels of Rac1GTP. Knock-down of HMHA1 significantly increases Rac1GTP loading compared to control cells. Data are mean values of two (for shHMHA1 #3) or three (for shHMHA1 #2) independent experiments. Error bars indicate SD. * p<0.05. (D) Jurkat cells transduced as indicated were lysed and RhoA-GTP levels were measured using a RhoA G-LISA. No significant differences in levels of RhoA-GTP loading were observed between control cells and cells treated with shRNAs against HMHA1. Data are mean values of four measurements of two independent experiments. Error bars indicate SD.

Next we investigated whether HMHA1 regulates RhoGTPase activity *in vivo*. First, we transfected HeLa cells with HMHA1 full-length (FL), the N-terminal region (N-term), and C-terminal constructs (C1-GAP and C1-GAPtail), that lack the BAR domain and measured Rac1GTP loading. Similar to our *in vitro* results (Figure 8C, D), full-length HMHA1 and the N-terminal region did not significantly affect Rac1GTP loading. However, both C1-GAP and C1-GAPtail drastically reduced Rac1GTP levels (Figure 9A) indicating that HMHA1 functions *in vivo* similar as *in vitro*.

Next, we analyzed whether HMHA1 can regulate RhoA-GTP loading *in vivo* using a RhoA G-LISA. As for Rac1GTP loading, HMHA1 C1-GAPtail reduces levels of RhoA-GTP compared to control cells (Figure 9B) indicating that *in vivo* HMHA1 can regulate activity of both Rac1 and RhoA. As HMHA1 is not endogenously expressed in HeLa cells we could not analyze Rac1- or RhoA GTP loading when HMHA1 levels are reduced by short interfering RNAs. As expression of HMHA1 is, under normal conditions, restricted to the hematopoietic system, we performed both a CRIB pull-down assay and a RhoA G-LISA using Jurkat T cells that express endogenous HMHA1. HMHA1 expression was reduced via lentiviral shRNA to HMHA1. As expected, knock-down of HMHA1 in Jurkat cells, using two shRNA constructs, significantly increased Rac1GTP loading (Figure 9C). Interestingly, in contrast to our results in HeLa cells, knock-down of HMHA1 does not significantly alter levels of RhoA-GTP in Jurkat cells (Figure 9D) suggesting that in these cells HMHA1 may function different than in HeLa cells. Although our *in vitro* data suggest that HMHA1 regulates Cdc42, we did not observe altered levels of Cdc42GTP in HeLa cells expressing HMHA1 C1-GAPtail (data now shown).

Finally, we performed extensive analysis of chemotaxis of Jurkat cells towards CXCL12. However, despite efficient reduction of HMHA1 levels in Jurkat cells using lentiviral shRNA expression, we observed no loss of chemotactic activity towards CXCL12. This could be due to residual HMHA1 expression, which is expressed to high levels in these cells (Figure 6) or to redundancy. Loss of HMHA1 could be functionally compensated by related RhoGAP proteins [18]. Similarly, loss of HMHA1 may increase Rac1 activity, but this may not be relevant for chemotaxis in these cells. This is not unprecedented, as it was shown for macrophages that neither Rac1 nor Rac2 were required for cell migration [34]. Related GTPases, such as RhoG, could control cell migration under these conditions [35]. To what extent such related GTPases are targeted by HMHA1, or related GAP proteins, remains to be investigated. Finally, it could be that the 2–2.5 fold-increased Rac1 activity in these cells (Figure 9C) neither impairs nor promotes CXCL12-induced motility, since CXCL12 also stimulates Rac1 activity. Thus, defining the exact role of HMHA1 in motility of lymphoid cells requires further analysis.

## Discussion

The human minor H antigen HMHA-1 has been widely studied in the context of human Stem Cell Transplantation (SCT) [36]. The minor H antigen HMHA-1 is a highly immunogenic nonameric peptide, encoded by the HMHA1 protein, and presented to the immune system in an HLA-restricted fashion. The minor H antigen HMHA-1 is expressed on all cells of the hematopoietic system as well as on solid epithelial tumors of most entities [4], [5]. Based on its extraordinary expression patterns, HMHA-1 is an ideal tumor target for Stem Cell based immunotherapy [36]. So much attention had been focused on the minor H antigen HMHA-1 in SCT, so little attention received the cell biological role of its encoding gene, i.e. HMHA1. Challenged by the expression and immunological characteristics of the minor H antigen HMHA-1, we made a first attempt to disclose the function of the HMHA1 gene that encodes the minor H antigen HMHA1. Since our previous analysis of the primary sequence of HMHA1 revealed that the protein encodes an N-terminal BAR domain followed by a C1 and a RhoGAP domain (Figure 1A), we focused on a potential role for HMHA1 in the regulation of RhoGTPases [6].

RhoGTPase signaling is tightly controlled. Indeed, aberrant signaling has often been linked to malignancies. GAPs terminate RhoGTPase activity as they increase the intrinsic hydrolysis rate of RhoGTPases [14]. Thus, RhoGAPs limit the duration and level of GTPase signaling output. In the present study, we identified HMHA1 as a novel RhoGAP. We found that HMHA1 shows high sequence homology with known GAPs such as GRAF1 and p50RhoGAP including the critical arginine finger in the catalytic domain. Furthermore, the model we generated of the HMHA1 RhoGAP domain in complex with RhoA suggests that HMHA1 is a RhoGAP. Our *in vitro* studies further supported this by showing that HMHA1 has GAP activity towards the RhoGTPases, Rac1, Cdc42, and RhoA. Moreover, the N-terminal BAR domain of HMHA1 acts as an autoinhibitory module for GAP function as full-length HMHA1 showed little GAP activity.

Based on these observations, HMHA1 can be positioned in a subfamily of structurally related proteins that comprise an N-terminal BAR domain followed by a RhoGAP domain[18]. Well-studied proteins belonging to this subfamily of BAR-GAPs are RICH1, OPHN1, SH3BP1, and GRAF1 [19]–[21], [37]. Similar to what we observed for HMHA1, the GAP function of these proteins is auto-inhibited by their N-terminal BAR domain. For OPHN1 and GRAF1, it was shown that the N-terminal BAR domain can interact with the GAP domain, inhibiting its function [26]. The mechanism by which the auto-inhibition in HMHA1 is released remains to be investigated. This most likely involves stimuli that target these BAR-GAPs to specific sites within the cell or induce protein-protein interactions, unfolding the protein and releasing the GAP domain from BAR-domain-mediated inhibition.

By regulating RhoGTPase activity, HMHA1 regulates the actin cytoskeleton. A C-terminal fragment of HMHA1, lacking the BAR domain, had strong effects on cell morphology. Similar effects were seen when mutants of SH3BP1 lacking its BAR domain were expressed [19]. Cells expressing the HMHA1 mutants lacking the N-terminus including the BAR domain show loss of F-Actin and focal adhesions. Moreover, these cells spread less compared to control cells. This phenotype is in line with our data showing that HMHA1 acts as a RhoGAP, because RhoGTPase activity is needed for proper cell adhesion and migration. Although we observe *in vitro* GAP activity towards Rac1, Cdc42, and RhoA, it could well be that *in vivo*, only a subset of these GTPases is subject to control by HMHA1. The dramatic phenotype induced by the HMHA1 C1-GAPtail construct was rescued by co-expressing constitutively active Rac1 but not Cdc42 suggesting that HMHA1 primarily inactivates Rac1 *in vivo*. It is well established that the small GTPase RhoA regulates stress fiber formation and focal adhesion turnover [38]. Our data indicate that HMHA1 regulates RhoA *in vitro* and *in vivo* although it was not possible to do the rescue experiments to further substantiate this. Our *in vivo* activity assays indicate that HMHA1 regulates Rac1GTP and RhoA-GTP loading in HeLa cells. In contrast, in Jurkat cells, HMHA1 only regulates the levels of Rac1GTP. These data further support the notion that Rac1 and RhoA are *in vivo* targets of HMHA1. Whether Cdc42 activity is regulated *in vivo* by HMHA1 requires further investigation.

In many epithelial cancers, a change in tissue architecture called epithelial-mesenchymal transition (EMT) occurs [39], [40]. This results in disruption of intercellular contacts and enhanced cell motility leading to the release of single cells from the epithelial tissue [40], [41]. RhoGTPases, and in particular Rac1 and RhoA, regulate epithelial cell-cell adhesion [42], [43]. Being a regulator of RhoGTPase output, abnormal HMHA1 expression in epithelial cells could cause EMT, tumor cell invasion and metastasis. Interestingly, although HMHA1 expression is restricted to the hematopoietic system under normal conditions [4], in many epithelial tumor cells HMHA1 gene expression was observed [5]. Moreover, minor H antigen HMHA-1-specific cytotoxic T cells eradicate solid epithelial tumors in an *in vivo* animal model [8]. Whether expression of HMHA-1 is causal for the generation of cancerous or metastasizing solid tumors remains to be investigated.

Understanding the role of HMHA1 in carcinogenesis might not only be relevant from a cell biological point of view. The capability of HMHA-1 specific immunotherapy to eradicate leukemia or solid tumors strongly depends on whether HMHA-1 is expressed on the earliest progenitor/stem cell from which the targeted leukemia or solid tumor can repopulate. Our data demonstrate for the first time that HMHA1 is not a coincidental "house keeping gene", but plays an important role for functions crucial for malignant cells. This may reduce the risk that HMHA1 protein expression will be silenced by the cancer cells to evade the attack by HMHA-1 specific CTLs. Remarkably, we could show that HMHA1 expression remains intact in residual primary leukemia cells even after successful treatment in our in vivo animal model [7]. Additional studies are needed to show a similarly persistent HMHA1 expression after HMHA-1 specific immunotherapy of solid tumors. Moreover, the fact that cytoskeletal remodeling and cell spreading are key functions of highly motile cells may well explain the hematopoietic restriction of the HMHA1 under normal conditions. Our previous studies showed that HMHA1 is highly expressed particularly in lymphocytes and in hematopoietic cells with phagocytic and/or antigen presenting capacity, i.e. monocytes, langerhans cells or dendritic cells [44]. HMHA1 knockdown experiments are needed to understand the effect of HMHA1 in inflammation.

In summary, we identified HMHA1 as a novel RhoGAP that regulates the actin cytoskeleton and cell spreading. As endogenous HMHA1 expression is normally limited to the hematopoietic system, future studies should be aimed at defining the role of HMHA1 in leukocytes in the context of actin remodeling, cell migration, phagocytosis and antigen presentation. Since HMHA1 expression is detected in several epithelial cancer cells, future studies should also address how HMHA1 is involved in the transformation and invasive behavior of these epithelial cells. Finally, HMHA1 might represent an excellent target for tumor therapy because healthy epithelium does not express HMHA1. The notion that HMHA1 indeed exerts GAP activity *in vivo*, supports further research in this area.

## Supporting Information

Figure S1 **HMHA1 is not involved in microtubule remodeling.** The effects of myc-tagged HMHA1 (and deletion constructs) on microtubule distribution was studied by Confocal Laser Scanning Microscopy using HeLa cells. Myc-tagged HMHA1 and microtubules were detected by immunostaining. Although cell morphology is clearly affected, no major effects on microtubule distribution are observed in HeLa cells expressing the indicated HMHA1 constructs. Scale bars, 10 µm.
(TIF)

Figure S2 **Immunostaining for endogenous HMHA1 and Rac1.** Jurkat cells were immunostained for endogenous HMHA1 and endogenous Rac1 and co-stained for F-actin using phalloidin. Because HMHA1 and Rac1 and F-actin showed a high level of colocalization (top panel), we confirmed lack of signal bleed-through by staining with only HMHA1 (second panel), only Rac1 (third panel) or phalloidin only (bottom panel) followed by analysis with identical settings as in Figure 6A. These analyses showed that there is no cross talk between the different channels, further confirming the colocalization analysis in Figure 6.
(TIF)

Figure S3 **HMHA1 directly interacts with RhoGTPase.** (A) Pull-down experiments using GST-EV, and GST-Rac1 loaded with GDP or GppNHP, a GTP analog that cannot be hydrolyzed, show that HMHA1 C1-GAPtail directly interacts with Rac1 preferably when Rac1 is in the active conformation. Association of purified C1-GAPtail was detected by immunoblotting (IB). Ponceau staining indicates equal loading of GST input. (B) Pull-down experiments with GST-Rac1 FL or ΔC, both loaded with either GDP or GppNHp, show that HMHA1 C1-GAPtail directly interacts with active Rac1, independent of the Rac1 hypervariable C-terminus. Association of purified HMHA1 C1-GAPtail was detected by immunoblotting. (C) Pull-down experiments using GST-Rac1 or GST-RhoA, both loaded with either GDP or GppNHp show that purified full-length HMHA1 directly interacts with both active Rac1 and RhoA. Association of purified HMHA1 was detected by immunoblotting.
(TIF)
