# Supplementary figures and images for: The Human Minor Histocompatibility Antigen1 Is a RhoGAP
Source: PLoS One. 2013 Sep 23;8(9):e73962. doi: 10.1371/journal.pone.0073962 (PMC3781157; doi:10.1371/journal.pone.0073962)

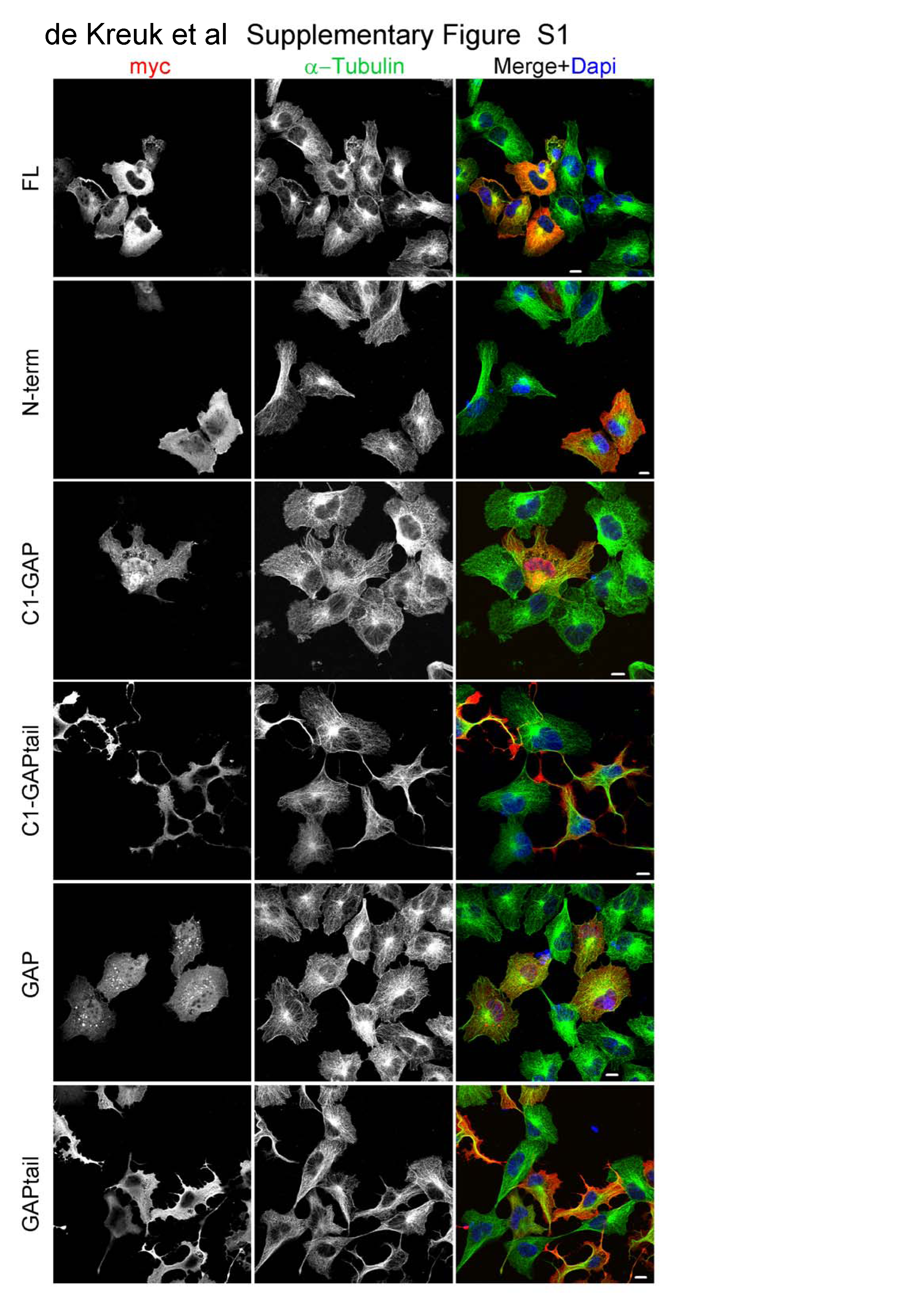

Supplement: Figure S1 — HMHA1 is not involved in microtubule remodeling. The effects of myc-tagged HMHA1 (and deletion constructs) on microtubule distribution was studied by Confocal Laser Scanning Microscopy using HeLa cells. Myc-tagged HMHA1 and microtubules were detected by immunostaining. Although cell morphology is clearly affected, no major effects on microtubule distribution are observed in HeLa cells expressing the indicated HMHA1 constructs. Scale bars, 10 µm. (TIF) [file pone.0073962.s001.tif]

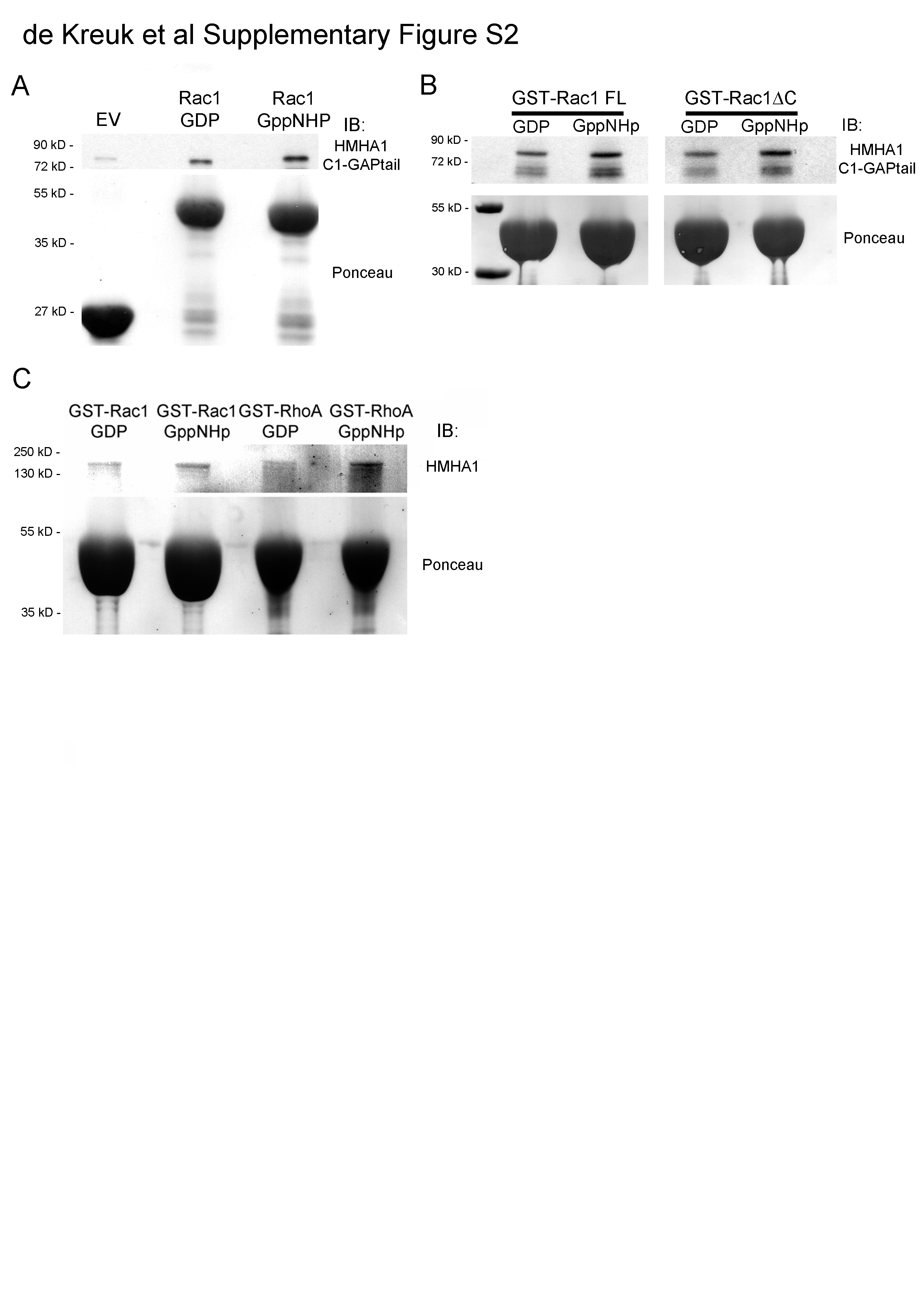

Supplement: Figure S2 — Immunostaining for endogenous HMHA1 and Rac1. Jurkat cells were immunostained for endogenous HMHA1 and endogenous Rac1 and co-stained for F-actin using phalloidin. Because HMHA1 and Rac1 and F-actin showed a high level of colocalization (top panel), we confirmed lack of signal bleed-through by staining with only HMHA1 (second panel), only Rac1 (third panel) or phalloidin only (bottom panel) followed by analysis with identical settings as in Figure 6A. These analyses showed that there is no cross talk between the different channels, further confirming the colocalization analysis in Figure 6. (TIF) [file pone.0073962.s002.tif]

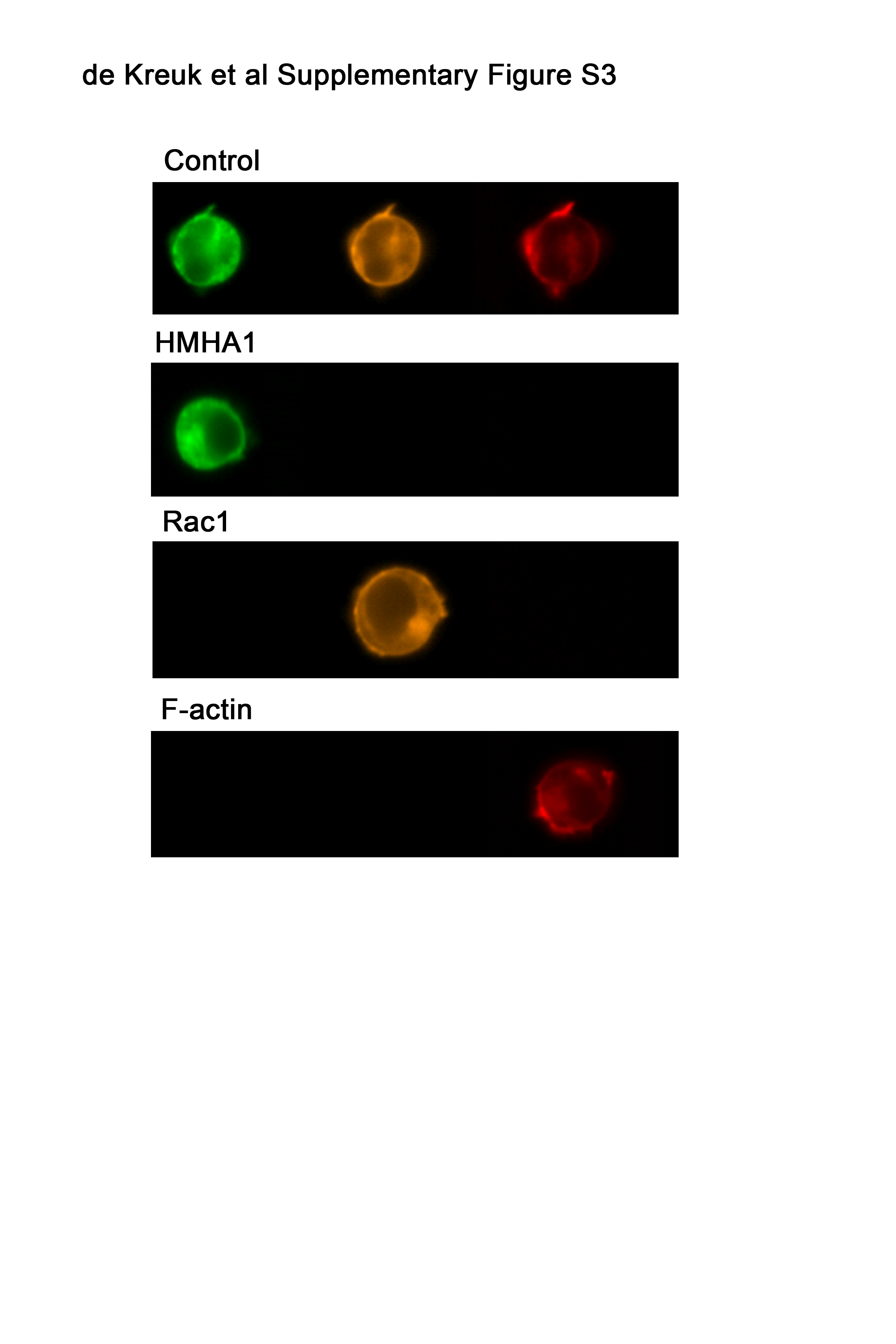

Supplement: Figure S3 — HMHA1 directly interacts with RhoGTPase. (A) Pull-down experiments using GST-EV, and GST-Rac1 loaded with GDP or GppNHP, a GTP analog that cannot be hydrolyzed, show that HMHA1 C1-GAPtail directly interacts with Rac1 preferably when Rac1 is in the active conformation. Association of purified C1-GAPtail was detected by immunoblotting (IB). Ponceau staining indicates equal loading of GST input. (B) Pull-down experiments with GST-Rac1 FL or ΔC, both loaded with either GDP or GppNHp, show that HMHA1 C1-GAPtail directly interacts with active Rac1, independent of the Rac1 hypervariable C-terminus. Association of purified HMHA1 C1-GAPtail was detected by immunoblotting. (C) Pull-down experiments using GST-Rac1 or GST-RhoA, both loaded with either GDP or GppNHp show that purified full-length HMHA1 directly interacts with both active Rac1 and RhoA. Association of purified HMHA1 was detected by immunoblotting. (TIF) [file pone.0073962.s003.tif]
